# Does Science Literacy Guarantee Resistance to Health Rumors? The Moderating Effect of Self-Efficacy of Science Literacy in the Relationship between Science Literacy and Rumor Belief

**DOI:** 10.3390/ijerph18052243

**Published:** 2021-02-24

**Authors:** Lingnan He, Yue Chen, Xiling Xiong, Xiqian Zou, Kaisheng Lai

**Affiliations:** 1 School of Communication and Design, Sun Yat-Sen University, Guangzhou 510006, China; heln3@mail.sysu.edu.cn (L.H.); cheny789@mail2.sysu.edu.cn (Y.C.); 2 Department of Psychology, Sun Yat-Sen University, Guangzhou 510006, China; 3 Guangdong Key Laboratory for Big Data Analysis and Simulation of Public Opinion, Guangzhou 510006, China; 4 School of Tourism Management, Sun Yat-Sen University, Zhuhai 519000, China; 5 School of Journalism and Communication, Jinan University, Guangzhou 510632, China; zouxiqian@stu2019.jnu.edu.cn

**Keywords:** science literacy, health rumor, self-efficacy

## Abstract

Health rumors not only incite unnecessary fears and skepticism, but may also cause individuals to refuse effective remedy and thus delay their treatment. Studies have found that health literacy may help the public identify the falsity of health rumors and avoid their negative impact. However, whether other types of literacy work in helping people disbelieve health rumors is still unknown. With a national survey in China (N = 1646), our study examined the effect of science literacy on rumor belief and further analyzed the moderating role of self-efficacy of science literacy in their relationship. Hierarchical regression analysis showed that science literacy significantly decreased the likelihood of people believing in health rumors, and moderator analysis showed that self-efficacy of science literacy plays a moderating role in this relationship; such that the relationship between science literacy and health rumor belief would be weakened if one′s self-efficacy of science literacy was low. This finding reveals that during campaigns to combat health rumors, improving and enhancing the self-efficacy of people′s science literacy is an effective way to prevent them from believing in health rumors. Our study highlights the benefits of science education in public health and the improvement of public science literacy.

## 1. Introduction

Rumors are unverified and instrumental in circulating information [1]. Nowadays, the internet is flooded with health rumors for the purpose of capturing people′s attention for health information [2,3]. In recent times, due to the outbreak of COVID-19 pandemic, the spread of health rumors is more prevalent than usual, which has led to a new phenomenon—infodemic. “Infodemic” refers to too much information, both correct and incorrect, making it difficult for people to find trustworthy sources of information, and may even harm people′s health [4]. This phenomenon needs to be combated along with the spread of COVID-19 [5].

Existing literature has proposed some effective measures to restrict the negative effects of health rumors, including strengthening the relevant law enforcement, spreading truthful information to balance the rumors, and enhancing the public′s ability to identify the authenticity of all kinds of information [6]. Researchers, importantly, have found that if more people can identify rumors, the spread of rumors in the social network would be contained [7]. Therefore, researchers have shown interest in the role of literacy in rumor-belief [8,9,10,11]. Moreover, literacy improvement is presented as a promising strategy for reducing the negative effects of rumors on individuals’ health decisions [9]. Based on different domains and emphases, a wide array of literacy concepts has been developed [10], such as media literacy, information literacy, news literacy, digital literacy, etc. Researchers have found that different literacies show different functions in combating fake news, and not every kind of literacy work is helping people identify misinformation [8]. Some studies found that specific literacy, such as health literacy [11], enables the public to deter the harmful effects of fake news, while another study found that media literacy did not safeguard against misinformation properly [8]. Instead, a more general and conceptual type of literacy, such as information literacy, enabled people to recognize and refute fake news online [8].

Previous studies have argued that only education that was directly addressing a specific misbelief, such as pseudoscience, led to a reduction of those beliefs, while unrelated general educational courses, such as classes on critical thinking and research methods, did not [12]. This phenomenon might be attributed to various confounding factors that were not accounted for in previous studies. Therefore, in our study, we drew on the self-efficacy theory [13], which states that one′s confidence in their own abilities can affect their performance; thus, we assumed a link between general literacy and health rumor belief (HRB). Evidence suggests that health literacy could stimulate people to verify the health information they see on social media before believing in it [14]; however, whether a more general type of literacy, such as science literacy, works well in helping people disbelieve rumors related to healthcare is still unknown.

**Figure 1:**
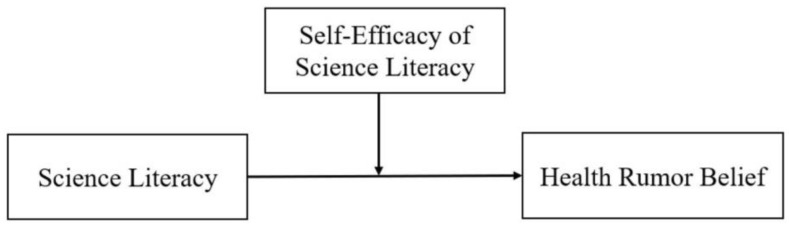
Hypothesized model of self-efficacy, science literacy, and rumors.

Our research seeks to answer three questions: (a) Does science literacy affect individuals′ health-based rumor belief? (b) What is the role of self-efficacy in such a dynamic? (c) When does self-efficacy become more important to this relationship? To answer these questions, we proposed a conceptual model, as illustrated by Figure 1, and empirically tested it with an online survey.

The contribution of this model is that it directly assesses the relationship between scientific literacy and rumor belief. Moreover, we aimed to explore the moderating role of self-efficacy. This research sought to enrich the public health literature by underscoring the importance of self-efficacy in improving individual scientific literacy through science education to improve people′s rumor identification abilities. We also sought to demonstrate how self-efficacy could help activate the power of science literacy on individuals′ rumor belief, in order to offer new insights on public health to halt individuals′ HRB.

### 1.1. Literature Review

#### 1.1.1. Damage of Health Rumors

Health organizations are dedicated to protect and improve the public′s health status; they repeatedly urge the public to follow the recommended instructions [15]. However, these efforts are hampered by health rumors. People′s belief in health rumors can trigger skepticism and resistance to the recommended medical behaviors, and they may refuse proper remedy and effective preventative behaviors [16]. Some might subscribe to a wrong way of treatment due to health rumors, and thus may face negative health consequences including death [17]. For example, health rumors claiming that vaccinations cause autism made many parents refuse immunizations for their children, which led to preventable diseases or deaths [16]. Health rumors are a threat to public health [18]; they hinder people’s engagement in desirable health behaviors and institutional efforts to manage public health [19]. Once people accept rumors, it becomes difficult to dispel their misconceptions [17]. In some cases, the effort for retracting misinformation can even backfire and increase misbelief [17], which makes it difficult for government and health organizations to refute health rumors and stop the harmful consequences of believing in them. Currently, the efforts to fight against COVID-19 are being compromised by the infodemic. Thus, making it imperative to study health rumors to reduce their imminent harm.

#### 1.1.2. Science Literacy

Science literacy is defined as “a broad understanding of the methods of science and a general knowledge of some of its specific content” [20]. It focuses on the technical knowledge of science. At the beginning of the 21st century, the call for science literacy education swept the world. Countries around the world established science education reforms emphasizing science literacy for children before high school [21] as its importance was paramount for modern society to function properly [22,23].

Evidence suggests that science literacy can help in eliminating superstition [24] and identifying misinformation on social media [25]. Promoting scientific literacy at the group level is beneficial to control rumor transmission [6]. Notably, a study found a negative correlation between science literacy and unwarranted beliefs [20] and rumors are often considered to be examples of unwarranted beliefs [25]. Scientists believe that science literacy helps people make appropriate health decisions [26]. Therefore, we propose the following hypothesis:

**Hypothesis** **1.** *Individuals with higher science literacy are more likely to identify health rumors.*

#### 1.1.3. Self-Efficacy Theory

Self-efficacy refers to perceived capabilities for performing actions at designated capacities [14]. It is often considered as the foundation of human accomplishments, and it can positively influence effort expenditure and task persistence, especially in the case of obstacles [27]. As it is a kind of a decision-making task, health rumor identification could be impacted by self-efficacy.

Previous findings suggest that self-efficacy had a moderating effect on the relationship between objective conditions and outcomes [28,29,30,31,32,33], such as strengthening the positive relation between personal skills and work performance [28]. Additionally, previous research has examined the differences between actual literacy and self-assessed literacy [29]. People′s self-ratings of their skills tend to increase with the increasing level of actual skill, though their self-assessment may not always be accurate [30]. Furthermore, studies have found a high self-assessment of skill enables people to perform better during a cognitive task [31].

Self-efficacy beliefs affect performance by influencing numerous psychological processes [32,33]. For example, individuals who have a strong belief that they can succeed in science tasks will be more likely to work hard to complete them successfully. Specifically, researchers posit that individuals′ belief in their ability to succeed in science tasks (e.g., reading scientific information) will influence their choices of science-related activities [33,34,35]. Alternatively, when those individuals who have weak self-efficacy beliefs related to science are confronted with the typical challenges involved in science, they will be more likely to give up and to experience stress [36]. This withdrawal from scientific tasks may lead to more uncertainty about information judgments, and thus may lead to believing in rumors [1]. Therefore, self-efficacy may serve as a moderator and increase the positive effect of science literacy on rumor identification.

To summarize, the aim of our study is to examine whether self-efficacy plays a moderating role in the relationship between science literacy and belief in rumors.

**Hypothesis** **2.** *Self-efficacy increases the positive effect of science literacy on health rumor identification as a moderator.*

## 2. Materials and Methods

### 2.1. Participants and Procedures

An online survey was conducted in Mainland China through the Tencent Questionnaire platform during May 2018. This platform belongs to the most popular social media company in China and has a large number of users. All participants were anonymous and volunteered to answer the survey without any extrinsic incentive. All the users′ IP addresses were recorded by the questionnaire system, and therefore the survey could only be accessed once from one device, which helped in negating the influence of bots. In total, we collected a sample of 1646 adults, which varied in demographic characteristics, such as age, gender, and income. Detailed sample characteristics are presented in Table 1.

In this survey, participants were first asked to read five rumors (which were identified to be false by a fact-checking website) [37] and indicate their belief for each one. Thereafter, they were asked to finish a 13-item test of basic science knowledge. Participants were then asked to rate their level of self-efficacy of science literacy from 1 to 10. Finally, they answered some demographic questions. The research was examined and approved by the Academic Ethics Committee of Department of Psychology, Sun Yat-Sen University.

### 2.2. Measurements

Health Rumor Belief (HRB). The questionnaire consisted of five rumors related to the health domain. The participants were asked to rate the extent of their belief in those rumors (1 = extreme doubt, 5 = extreme belief) (e.g., “drinking overnight boiled water causes cancer”). To better identify those participants who were more likely believe health rumors, responses were dichotomized following the methodology of previous studies [38]. Only participants who disbelieved the health rumors and answered 1 (extreme doubt) or 2 (doubt) in response to either of the five rumors were identified as not-believing (0), being immune to health rumors; otherwise, those who answered 3 (maybe), 4 (believe), or 5 (extreme belief) in any of the five rumors were identified as believing (1), misbelieving in health rumors.

Science Literacy (SL). The measurement of science literacy is taken from the Chinese citizen science literacy survey [39], which was adapted to the survey from Science & Engineering Indicators [40]. This survey contains 13 items that test basic science knowledge where participants are required to judge whether they are true or false. A few examples of these items are “All radiation is caused by humans’ activities” or “Smoking can cause lung cancer”. This survey has been widely used [22,41] and has good validity. Participants got one point for a correct answer and all the points were added to get a total score (which could range from 0–13 points). The higher the total score, the higher was one’s science literacy.

Self-efficacy of science literacy (SSL). Following the measurement of self-efficacy of previous studies [42,43], SSL was measured with a single item that asked participants to evaluate their own science literacy (1 = extremely low, 5 = extremely high).

Control variables. Previous research has illustrated that internet use [44] was a significant predictor of people’s likelihood to believe in rumors. Therefore, we included this variable along with other traditional demographic [45] controls in our analyses. As for internet use, participants were asked how many hours in a day, on average, do they spend surfing the internet. Other demographic variables, including gender, age, education, and income, also served as control variables and were added in the regression analysis as continuous variables. In the descriptive statistics results, we recoded age into groups in order to compare results across different age subsets.

## 3. Results

### 3.1. Characteristics of Groups Believing and Not-Believing Health Rumors

Among the 1646 participants (73.4% male, 26.6% female, mean age 29.26), 1238 (75.2%) succeeded in identifying all five rumors, whereas 408 (24.8%) failed to identify some or all of the rumors. Table 1 summarizes the demographic characteristics of the not-believing and believing groups. The two groups had no significant difference in gender (χ^2^ = 0.024, d.f. = 1, *p* = 0.898), but age (χ^2^ = 23.876, d.f. = 3, *p* < 0.001), education (χ^2^ = 33.970, d.f. = 4, *p* < 0.001), and income (χ^2^ = 39.877, d.f. = 7, *p* < 0.001) were found to be a contributing factors. Older, lesser educated, and low-income participants were more likely to believe health rumors.

### 3.2. Science Literacy and HRB

Results from logistic regression analysis indicate that science literacy (M = 10.15, SD = 1.69) was negatively associated with HRB (OR = 0.872, 95% CI: 0.814–0.935) even after controlling for gender, age, social media use, education, and income (see Table 2). It indicated that the higher one′s science literacy is, the lesser is the possibility of believing in health rumors.

In addition, education and income show a positive relationship with HRB, indicating that individuals with less education or lower income were more likely to believing in rumors. In contrast, age was negatively associated with HRB, indicating that older individuals were more likely to believing rumors. Last, gender and internet use had no significant relationship with HRB.

### 3.3. Moderation Analysis

**Figure 2:**
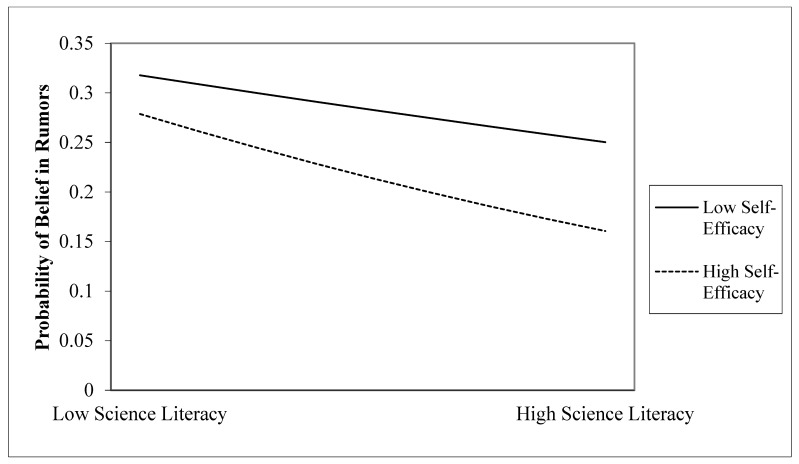
Interaction of science literacy and self-efficacy of science literacy.

We used PROCESS model 1 [46] to apply the moderating analysis and the results are shown in Table 3. The interaction of SL and SSL (M = 3.08, SD = 1.04) was significant (b = −0.06, *p* < 0.05), which showed that SL predicted lower HRB when their SSL was high (1 SD above the mean), b = −0.20, *p* < 0.001. Although the coefficient of SL on HRB was negative when their SSL was low (1 SD below the mean), the influence of SL was insignificant, b = −0.07, *p* = 0.115 (Figure 2). It indicated that self-efficacy of science literacy has a moderating effect on science literacy and HRB. In other words, the buffering effect of SL on HRB was only effective for individuals with high SSL. In addition, note that males have a higher SSL than females, F (1, 1835) = 31.028, *p* < 0.001. However, as shown in Table 3, when gender is used as a control variable, there is no significant effect; thus, we did not conduct an in-depth analysis for gender effect.

## 4. Discussion

The goal of this investigation was to examine the impact of science literacy on individuals’ HRB from a self-efficacy perspective. People’s science literacy was negatively associated with the likelihood of believing in rumors, and the buffering effect of science literacy was only effective for individuals with high self-efficacy. These results suggest that science literacy has a protective effect for people′s HRB, and more importantly, that it is more effective when individuals′ self-efficacy of science literacy is high. It suggests that science education might be protective for different reasons than previously thought. Both theoretical and practical implications are presented below.

### 4.1. Theoretical Implications

Health rumors not only cause the spread of unnecessary fears and conspiracies [47], but also may make individuals handle their illness improperly, which may delay their treatment and other negative health consequences [10]. We showed that high science literacy could protect people from falling into the trap of health rumors, unlike the results of previous studies that suggested science literacy was not a guarantee for protecting against unwarranted belief [48,49]. A study by Dyer and Hall [12] argued that education specifically directed to a specific field could produce a reduction of incorrect beliefs in that particular field. However, we found general science literacy is effective for health rumor identification in the general public. As previous research has suggested [22,50], the function of science literacy is not limited to the particularity of a certain field.

This paradoxical phenomenon is articulated in the present research by drawing on the self-efficacy theory [13]. Our findings provide new insights to the aforementioned discrepancy by illustrating that the protective effect of science literacy is contingent on whether individuals′ self-efficacy of science literacy is high. To this end, this research improves our understanding of the theoretical conceptions pertaining to science literacy and HRB. Specifically, it offers nuances to self-efficacy theory by demonstrating the moderating effect of self-efficacy of science literacy on disbelieving health rumors.

### 4.2. Practical Implications

Our results show that the benefits of learning scientific knowledge may be helpful for rumor identification. When conducting anti-health rumor campaigns, healthcare institutions and practitioners should consider the effectiveness of science literacy. Investment in improving citizens’ science literacy is important as it can help dispel myths and rumors, and decrease expenditure incurred towards controlling panic caused by health rumors. It is necessary to explore more ways to encourage citizens to receive science education in schools and motivate them to read scientific material daily.

Furthermore, we found the protective effect of science literacy only affects those individuals who have high self-efficacy of science literacy. This finding was consistent with the studies that found self-efficacy to be a moderator in improving individual’s general performance [51]. Thus, it is suggested that enhancing publics’ confidence in science literacy is one of the ways for healthcare practitioners to combat health rumors on the internet.

## 5. Limitations

The present study has some limitations. First, there may be a sampling bias as we collected the data via online social media. The sample may be more representative of a younger population, with more education, and more hours of daily internet use. Second, the self-reporting method for measuring HRB may be biased by social desirability [52]. Future studies could explore a more objective method to measure HRB. Finally, our research is based on a Chinese sample. The possible consequences of differences in self-efficacy of different cultures may also vary. The impact of cultural factors deserves further exploration in future research.

## 6. Conclusions

In conclusion, our study showed two main findings. First, to the best of our knowledge, this is the first study to examine the role of science literacy in identifying health rumors, and we found that increasing science literacy reduces the likelihood of people believing in health rumors. Second, self-efficacy of science education plays a moderating role in the relationship between scientific knowledge and rumor belief. We also found that scientific knowledge was only effective for individuals with high self-efficacy of science literacy. When healthcare practitioners are making efforts to advance public science literacy levels, they should simultaneously pay attention to enhancing the public′s self-efficacy levels in identifying health rumors.

## Figures and Tables

**Table 1:** Demographic characteristics (% of column total) and statistical tests between groups.

Demographic	Rumor Belief			
	Total	Not-Believing (n = 890)	Believing (n = 946)	χ^2^
**Gender**				0.438
Male	1195	900 (75.3)	676 (24.7)	
Female	451	295 (74.9)	270 (25.1)	
**Age (in years)**				23.876 ***
18–29	856	677 (79.1)	179 (20.9)	
30–39	490	365 (74.5)	125 (25.5)	
40–49	229	153 (66.8)	76 (33.2)	
50–69	71	43 (60.6)	28 (39.4)	
**Education**				29.895 ***
less than elementary school	157	92 (58.6)	65 (41.4)	
middle school	370	270 (73.0)	100 (27.0)	
some college	389	291 (74.8)	98 (25.2)	
Bachelor′s degree	604	486 (80.5)	118 (19.5)	
Master′s degree or higher	126	99 (78.6)	27 (21.4)	
**Income**				42.829 ***
below 2000 Ұ	44	35 (79.5)	9 (20.5)	
2001–6000 Ұ	323	205 (63.5)	118 (36.5)	
6001–10,000 Ұ	405	306 (75.6)	99 (24.4)	
10,001–15,000 Ұ	362	278 (76.8)	84 (23.2)	
15,001–30,000 Ұ	293	242 (82.6)	51 (17.4)	
30,001–45,000 Ұ	96	73 (76.0)	23 (24.0)	
45,001–60,000 Ұ	40	37 (92.5)	3 (7.5)	
above 60,000 Ұ	83	62 (74.7)	21 (25.3)	

*** *p* < 0.001.

**Table 2:** Logistic Regression Analyses.

Variable	B	S.E.	Wald	OR	*EXP(B)* 95% CI	
					Lower	Upper
Science Literacy	−0.137	0.036	14.826	0.872 ***	0.814	0.935
gender	−0.022	0.134	0.026	0.978	0.753	1.272
age	0.029	0.006	21.873	1.029 ***	1.017	1.041
social media use	0.004	0.019	0.056	1.004	0.968	1.042
education	−0.124	0.057	4.830	0.883 *	0.790	0.987
income	−0.089	0.038	5.436	0.915 *	0.848	0.986
Constant	0.045	0.431	0.011	1.046		

* *p* < 0.05, *** *p* < 0.001.

**Table 3:** Interaction of science literacy and self-efficacy of science literacy.

Model	B	SE	Z
constant	−1.15	0.06	−18.88 **
Science Literacy	−0.23	0.06	−3.75 **
Self-efficacy of science literacy	−0.16	0.07	−2.44 *
SL x SSL	−0.11	0.06	−1.99 *
gender	−0.03	0.06	−0.49
age	0.29	0.06	4.97 **
education	−0.09	0.07	−1.39
income	−0.14	0.06	−2.14 *
social media use	0.02	0.06	0.36

* *p* < 0.05, ** *p* < 0.01.

## Data Availability

The data presented in this study are available on request from the corresponding author.

## References

[1] DiFonzo N., Bordia P. (2007). Rumor Psychology: Social and Organizational Approaches.

[2] Wu K., Yang S., Zhu K.Q. False Rumors Detection on Sina Weibo by Propagation Structures. Proceedings of the 2015 IEEE 31st International Conference on Data Engineering.

[3] Sicilia R., Lo Giudice S.L., Pei Y., Pechenizkiy M., Soda P. (2018). Twitter Rumour Detection in the Health Domain. Expert Syst. Appl..

[4] WHO WHO Benin Goes on Digital Offensive against COVID-19. https://www.who.int/news-room/feature-stories/detail/benin-goes-on-digital-offensive-against-covid-19.

[5] WHO WHO Countering Misinformation about COVID-19. https://www.who.int/news-room/feature-stories/detail/countering-misinformation-about-covid-19.

[6] Zhou X., Feng H. (2019). Research on the Prevention and Control of the Internet Rumor from the Perspective of the Self-Media. J. Comput. Commun..

[7] Hu Y., Pan Q., Hou W., He M. (2018). Rumor Spreading Model Considering the Proportion of Wisemen in the Crowd. Phys. A Stat. Mech. Appl..

[8] Jones-Jang S.M., Mortensen T., Liu J. (2021). Does Media Literacy Help Identification of Fake News? Information Literacy Helps, but Other Literacies Don’t. Am. Behav. Sci..

[9] Qi M., Ding L. (2018). The Education Strategy Analysis of University Network Literacy Based on Internet Rumors. Educ. J..

[10] DeWalt D.A., Berkman N.D., Sheridan S., Lohr K.N., Pignone M.P. (2004). Literacy and Health Outcomes: A Systematic Review of the Literature. J. Gen. Intern. Med..

[11] Oh H.J., Lee H. (2019). When Do People Verify and Share Health Rumors on Social Media? The Effects of Message Importance, Health Anxiety, and Health Literacy. J. Health Commun..

[12] Dyer K.D., Hall R.E. (2019). Effect of Critical Thinking Education on Epistemically Unwarranted Beliefs in College Students. Res. High. Educ..

[13] Bandura A., Adams N.E. (1977). Analysis of Self-Efficacy Theory of Behavioral Change. Cognit. Ther. Res..

[14] Qi J., Banerjee S., Chua A. Analyzing Medical Personnel’s Perceptions of Online Health Rumors. Proceedings of the International Multiconference of Engineers and Computer Scientists.

[15] Bode L., Vraga E.K. (2018). See Something, Say Something: Correction of Global Health Misinformation on Social Media. Health Commun..

[16] Jolley D., Douglas K.M. (2014). The Effects of Anti-Vaccine Conspiracy Theories on Vaccination Intentions. PLoS ONE.

[17] Lewandowsky S., Ecker U.K., Seifert C.M., Schwarz N., Cook J. (2012). Misinformation and Its Correction: Continued Influence and Successful Debiasing. Psychol. Sci. Public Interest.

[18] Tan A.S.L., Lee C.-J., Chae J. (2015). Exposure to Health (Mis) Information: Lagged Effects on Young Adults’ Health Behaviors and Potential Pathways. J. Commun..

[19] DiFonzo N. (2013). Rumour Research Can Douse Digital Wildfires. Nature.

[20] Fasce A., Picó A. (2019). Science as a Vaccine: The Relation between Scientific Literacy and Unwarranted Beliefs. Sci. Educ..

[21] Godin B., Gingras Y. (2000). What Is Scientific and Technological Culture and How Is It Measured? A Multidimensional Model. Public Underst. Sci..

[22] Liu X. (2009). Beyond Science Literacy: Science and the Public. Int. J. Environ. Sci. Educ..

[23] Miller J.D. (2002). Civic Scientific Literacy: A Necessity in the 21st Century. FAS Pub Interest Rep..

[24] Turiman P., Omar J., Daud A.M., Osman K. (2012). Fostering the 21st Century Skills Through Scientific Literacy and Science Process Skills. Procedia Soc. Behav. Sci..

[25] Chou W.-Y.S., Oh A., Klein W.M.P. (2018). Addressing Health-Related Misinformation on Social Media. JAMA.

[26] Shen B.S. (1975). Science Literacy and the Public Understanding of Science. Communication of Scientific Information.

[27] Eastin M.S., LaRose R. (2000). Internet Self-Efficacy and the Psychology of the Digital Divide. J. Comput. Mediat. Commun..

[28] Speier C., Frese M. (1997). Generalized Self Efficacy as a Mediator and Moderator Between Control and Complexity at Work and Personal Initiative: A Longitudinal Field Study in East Germany. Hum. Perform..

[29] Mahmood K. (2016). Do People Overestimate Their Information Literacy Skills? A Systematic Review of Empirical Evidence on the Dunning-Kruger Effect. Commun. Inf. Lit..

[30] Cheema J.R., Skultety L.S. (2017). Self-Efficacy and Literacy: A Paired Difference Approach to Estimation of Over-/under-confidence in Mathematics- And Science-Related Tasks. Educ. Psychol..

[31] Bouffard-Bouchard T. (1990). Influence of Self-Efficacy on Performance in a Cognitive Task. J. Soc. Psychol..

[32] Bandura A. (1986). Social Foundations of Thought and Action: A Social Cognitive Theory.

[33] Bandura A. (1997). Self-Efficacy: The Exercise of Control.

[34] Britner S.L., Pajares F. (2001). Self-efficacy beliefs, motivation, race, and gender in middle school science. J. Women Minor. Sci. Eng..

[35] Zeldin A.L., Pajares F. (2000). Against the odds: Self-efficacy beliefs of women in mathematical, scientific, and technological careers. Am. Educ. Res. J..

[36] Britner S.L., Pajares F. (2006). Sources of science self-efficacy beliefs of middle school students. J. Res. Sci. Teach..

[37] Sohu Rumor-Vulnerable Group Report. http://www.sohu.com/a/135037975_664487.

[38] Achterhof R., Huntjens R.J.C., Meewisse M.L., Kiers H.A.L. (2019). Assessing the Application of Latent Class and Latent Profile Analysis for Evaluating the Construct Validity of Complex Posttraumatic Stress Disorder: Cautions and Limitations. Eur. J. Psychotraumatol..

[39] China Association of Science and Technology (2004). Survey Report on Chinese Public Scientific Literacy in 2001.

[40] National Science Board (États-Unis) (2002). Science & Engineering Indicators 2002.

[41] Drummond C., Fischhoff B. (2017). Individuals with greater science literacy and education have more polarized beliefs on controversial science topics. Proc. Natl. Acad. Sci. USA.

[42] Sutton S., Marsh A., Matheson J. (1987). Explaining Smokers’ Decisions to Stop: Test of an Expectancy-Value Approach. Soc. Behav..

[43] Gunnarsdottir T., Njardvik U., Olafsdottir A.S., Craighead L.W., Bjarnason R. (2011). The Role of Parental Motivation in Family-Based Treatment for Childhood Obesity. Obesity.

[44] He L., Yang H., Xiong X., Lai K. (2019). Online Rumor Transmission among Younger and Older Adults. Sage Open.

[45] Lai K., Xiong X., Jiang X., Sun M., He L. (2020). Who Falls for Rumor? Influence of Personality Traits on False Rumor Belief. Pers. Individ. Dif..

[46] Hayes A.F. (2017). Introduction to Mediation, Moderation, and Conditional Process Analysis: A Regression-Based Approach.

[47] Anderson J., Rainie L. (2017). The Future of Truth and Misinformation Online.

[48] Dentith M. (2013). Have You Heard? The Rumor as Reliable. Rumor and Communication in Asia in the Internet Age.

[49] Majima Y. (2015). Belief in Pseudoscience, Cognitive Style and Science Literacy. Appl. Cognit. Psychol..

[50] Feinstein N. (2011). Salvaging science literacy. Sci. Educ..

[51] Chen G., Casper W.J., Cortina J.M. (2001). The Roles of Self-Efficacy and Task Complexity in the Relationships Among Cognitive Ability, Conscientiousness, and Work-Related Performance: A Meta-Analytic Examination. Hum. Perform..

[52] Fisher R.J. (1993). Social desirability bias and the validity of indirect questioning. J. Consum. Res..

